# Treatment-seeking threshold and accessibility of psychiatric outpatient services in Switzerland: the relationship with stigma and self-esteem

**DOI:** 10.3389/fpsyt.2024.1377971

**Published:** 2024-04-12

**Authors:** Janina Billian, Lukas Imfeld, Carl B. Roth, Julian Moeller, Undine E. Lang, Christian G. Huber

**Affiliations:** ^1^ Universitäre Psychiatrische Kliniken (UPK) Basel, Klinik für Erwachsene, University of Basel, Basel, Switzerland; ^2^ Faculty of Psychology, Division of Clinical Psychology and Epidemiology, University of Basel, Basel, Switzerland; ^3^ Institute for Evaluation Research, Universitäre Psychiatrische Kliniken (UPK) Basel, University of Basel, Basel, Switzerland

**Keywords:** social stigma, stereotyping, patient satisfaction, self report, mental health, community mental health services, ambulatory care, health services research

## Abstract

Perceived stigmatization and low self-esteem are linked to poorer mental health outcomes, but their impact on treatment-seeking thresholds and the importance of outpatient service location remain unclear. The study included 525 outpatients of the University Psychiatric Clinic (UPK) Basel, Switzerland, of whom 346 were treated at inner city services and 179 at services located on the main site of the UPK at the outer city limits. Perceived discrimination and devaluation (PDD), self-esteem (SE), treatment-seeking threshold (TST), and accessibility were measured via a self-reported questionnaire. The PDD consisted of 12 items evaluating beliefs about the level of stigma towards individuals with mental illness in the general population on a 5-point Likert scale. SE, TST and accessibility were assessed through single-item 7-point Likert scales. PDD and SE were positively correlated (*p* < 0.001), suggesting that lower perceived stigma was linked to higher self-esteem, and were not associated with TST. The relationship between PDD and SE remained consistent after controlling for age, gender, and nationality. Age was negatively correlated with TST (*p* = 0.022), while gender did not significantly influence any of the variables. There was little variation regarding PDD, with emergencies at the site of the psychiatric clinic and substance use disorder (SUD) patients reporting higher levels of stigmatization. Emergency patients and those with SUD and personality disorder reported the lowest SE ratings. TST showed a broad range and was highest for emergency services and transcultural psychiatry patients. Differences in accessibility were mainly linked to the location, with outpatient service users in the inner city reporting better accessibility (*p* < 0.001) and higher SE (*p* = 0.009). In comparison to patients using services with planned contacts only, patients in emergency settings differed by higher TST (*p* = 0.018) and better ratings of accessibility (*p* = 0.004). In conclusion, there was a relevant amount of stigmatization, impaired self-esteem, and, for some outpatient services, high thresholds to seek treatment. Future research should explore other factors influencing TST. The findings highlight the need to address stigmatization and accessibility when planning mental health services.

## Background

To this day, suffering from a mental illness means a double burden for those affected due to the illness itself and the stigmatization of mentally ill people (1, 2). Despite the availability of effective treatments, a substantial number of individuals with mental illness continue to experience barriers in seeking and receiving appropriate care (3–5). Only about one third of individuals with mental illnesses actually seek mental health treatment (6).

One critical factor contributing to this treatment-seeking threshold is perceived stigma of mental illness, including both what an individual thinks most people believe about the stigmatized group in general and how the individual thinks society views him or her personally as a member of the stigmatized group (7). Negative and discriminatory prejudices toward people with mental illness that are frequently endorsed in society include perceptions of decreased trustworthiness and professional skills (8). Perception of public stigma is seen as the initial step toward developing self-stigma, which involves internalizing negative beliefs about oneself due to one’s mental health condition (8–14). At the same time, perceived stigma represents a major source of maintaining self-stigma (9, 15).

Perceived public stigma and self-stigma have been found to impact attitudes toward seeking psychological help (16–18). These factors also influence perceived need for help and the desire to self-manage mental health issues, potentially delaying or preventing help-seeking behavior (18–20).

Being an important barrier affecting an individual’s decision to seek care for mental health problems, perceived and self-stigma have been linked to adverse outcomes, including worsened mental health symptoms, treatment avoidance, poorer treatment adherence and higher suicide risk (21–25).

Individuals who internalize negative stereotypes about mental illness may experience feelings of shame, guilt, hopelessness, decreased self-esteem and lower self-efficacy, impacting their quality of life (15, 26–29). Perceived and self-stigma may lower an individual’s self-esteem by labeling themselves as socially unacceptable when seeking psychological help (16). In contrast, high self-esteem may play a significant role in mitigating the impact of perceived stigma on an individual’s well-being and improving motivation to seek treatment (15, 30, 31). In our study, we therefore expect to find significant correlations between perceived discrimination and devaluation (PDD), self-esteem (SE), and treatment-seeking threshold (TST).

While the link between mental illness stigma, self-esteem, and treatment-seeking threshold has gained considerable empirical attention, relatively less focus has been directed towards investigating the potential influence of the accessibility of psychiatric outpatient treatment sites in this context. Accessibility refers to the extent to which mental health services are readily available, physically reachable, affordable, and socially acceptable to individuals in need. Several factors can contribute to treatment site accessibility, including proximity, transportation options, cost, and cultural acceptability. Often, outpatient services are strategically placed at highly frequented and easy to reach local venues (e.g., in the city center or in proximity of a train station) to increase accessibility. On the other hand, economic considerations favor integrating integration of outpatient services with inpatient mental health clinics, which are often not optimized for accessibility and tend to be more stigmatized (32–34). For example, in the canton of Basel, the Psychiatric University Clinic operates multiple specialized outpatient services, some of which are within the inpatient mental health clinic perimeter, while others are strategically placed within the inner city limits. However, it is unclear if these different settings address patient populations with different help-seeking thresholds, and how they influence perceived stigma and self-esteem. Thus, regarding the potential impact of accessibility, we will explore the hypothesis that patients receiving treatment at psychiatric outpatient services in the inner city exhibit significantly different levels of perceived discrimination and devaluation (PDD), self-esteem (SE), treatment-seeking threshold (TST), and accessibility compared to the main site of the psychiatric university hospital at the outer city limits.

Other factors that affect the stigmatization of patients are the diagnosis and the type of service sought. Seeking help at psychiatric emergency services, often associated with severe mental illness, aggressive and self-harming behavior, can lead to heightened stigmatization (35). Thus, we will investigate whether the type of contact (planned contacts only versus unplanned emergency contacts) has a significant impact on perceived discrimination and devaluation (PDD), self-esteem (SE), and treatment-seeking threshold (TST).

Patients with substance use disorders, psychotic illnesses and personality disorders often face discrimination and devaluation due to prevailing societal prejudices against these specific conditions (36–39). For instance, patients with psychotic illnesses are seen as more dangerous and unpredictable compared to other mental illnesses (40, 41). Individuals with personality disorders are perceived as untreatable, manipulative and challenging in relationships (42, 43). Addiction disorders are frequently assumed to be self-inflicted resulting from a lack of discipline and are also more often associated with self-neglect and criminal behavior (37, 44, 45). Research has demonstrated that these particularly stigmatized patient groups have lower self-esteem compared to other mental health conditions (39, 46–49). Thus, regarding the treatment focus of the psychiatric outpatient service, we assume that there will be significant differences in perceived discrimination and devaluation (PDD), self-esteem (SE), and treatment-seeking threshold (TST).

Furthermore, gender, age, and cultural factors such as nationality and ethnicity can influence an individual’s experience of stigma and treatment-seeking behavior. According to previous studies, males are more likely to experience both public and self-stigma associated with psychological help-seeking in comparison to females, and women tend to show fewer stigmatizing attitudes (50–53). Aging, for instance, may influence perceived stigma and willingness to seek treatment, with older individuals showing lower stigma scores and more positive attitudes toward help-seeking (54). Ethnic minorities and migrant populations may face increased stigma and barriers to seeking help (55–57). At the same time, these groups seem to be more vulnerable to mental disorders, with a higher prevalence compared to the general population (58, 59). Thus, we hypothesize that the gender, age, and nationality of the participants have an influence on perceived discrimination and devaluation (PDD), self-esteem (SE), and treatment-seeking threshold (TST).

### Aims of the study

The current paper aims to examine if psychiatric outpatient treatment sites are addressing different patient groups regarding perceived stigma, self-esteem, and treatment-seeking threshold depending on their accessibility, and to explore the relationship between perceived stigma, self-esteem, and treatment-seeking threshold.

## Methods

**Figure 1:**
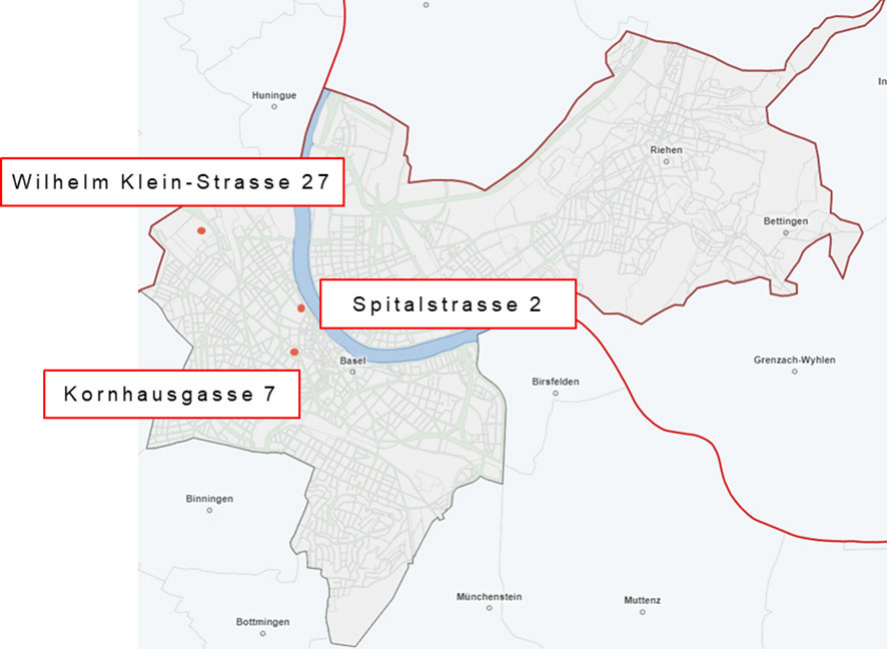
Locations of psychiatric outpatient services of the UPK Basel. Source: Statistical Office of the Canton of Basel-Stadt (Statistisches Amt des Kantons Basel-Stadt) https://www.basleratlas.ch.

The University Psychiatric Clinic (UPK) Basel is the only psychiatric university clinic in Northwestern Switzerland and serves a catchment area of about 200,000 persons in the canton of Basel-Stadt and the surrounding area. It provides psychiatric emergency care and basic psychiatric diagnostics and treatment for this population, but also maintains highly specialized services (e.g., for non-organic sleep disorders) for a larger catchment area. In 2018, 14 outpatient treatment services were available at the UPK Basel. Some of the services were located at the main site of the psychiatric clinic (Wilhelm Klein-Strasse 27, CH-4056 Basel) at the outer city limits, some were located within the inner city limits (Kornhausgasse 7, CH-4051 Basel), and one outpatient service was located within the inner city limits directly at the somatic university hospital (Spitalstrasse 2, CH-4056 Basel). **Figure 1** gives an overview on these geographic locations.

The UPK Basel routinely conducts anonymous patient satisfaction surveys as part of its quality management procedures. For the current paper, data from the patient survey conducted in 2018 were available. No ethics committee vote was necessary for the analysis and publication of this anonymously collected routine quality management data. This was confirmed by the responsible ethics committee (Ethics Committee of Northwestern Switzerland; EKNZ; Req-2023-01405).

### Participants and procedures

All patients who had at least one contact with a psychiatric outpatient service at the UPK Basel between 26^th^ March 2018 and 25^th^ June 2018 (cut-off date of the survey) and had a place of residence in Switzerland were invited to participate. A total of 2,203 patients were sent self-report questionnaires via standard mail within Switzerland. The questionnaires were provided in German. Due to organizational reasons, the questionnaires were not sent abroad. Participants were informed about the purpose and methodology of the survey and were assured of the confidentiality and anonymity of their responses. There was no time limit imposed on completing the questionnaires. A stamped return envelope was enclosed.

Data entry was performed by the Institute for Evaluation Research, Basel, a separate entity from the UPK Basel, to further ensure confidentiality. Paper questionnaires were scanned, data was converted into an electronic format, checked for plausibility and, if necessary, post-processed using Remark Office OMR, V8.0 (60). The data entry process involved assigning numerical codes to responses, thereby assuring anonymity of the participants, and then double-checking for data accuracy and completeness.

### Assessments

Perceived stigma was measured using the Perceived Discrimination Devaluation Scale (PDD), developed by Link (8), which consists of twelve items measuring respondents’ beliefs about the extent to which ‘most people’ would discriminate against and devalue individuals with a history of psychiatric treatment. The PDD, also known as ‘stereotype awareness’, evaluates individuals’ recognition of the negative views held by the general population towards those with mental illness (15).

Responses indicate the level of agreement with each statement, rated on a 5-point Likert scale ranging from 1 (“statement does not apply”) to 5 (“statement applies fully”). A low level of perceived stigma against people with mental illness is indicated by agreement with six positively poled items (e.g. “Most people would treat a former mental patient just as they would treat anyone.”) and disagreement with six negatively poled ones (e.g. “Most people think less of a person who has been in a mental hospital.”). The scale demonstrated sufficient global internal consistency of α = 0.84 (61).

For the purpose of this study, the German translation of the PDD was used (62). To facilitate statistical analysis, the six negatively worded items of the PDD were reversed so that higher values corresponded to lower perceived stigma. Then, a total PDD mean score was calculated by summing the values of all twelve items and dividing by twelve. As a result of reversing the negatively worded items, the final PDD mean score ranged from 1 (strongly perceived stigma) to 5 (low perceived stigma).

To assess self-esteem, the Single-Item Self-Esteem Scale (63) was utilized. Participants were instructed to rate their agreement with the statement “I have high self-esteem” on a 7-point Likert scale ranging from 1 (Not very true of me) to 7 (Very true of me). The Single-Item Self-Esteem Scale offers a test-retest reliability of 0.75 (63) according to the Heise procedure (64).

Treatment-seeking threshold (TST) was self-reported using a single item (“I had inhibitions about seeking treatment at the outpatient service.”) 7-point Likert scale ranging from 1 to 7 with higher values indicating a higher threshold. The item referred to the first contact to the outpatient service.

The accessibility of the treatment site was equally assessed through a self-report single item (“The outpatient treatment service was easily accessible for me.”) on a 7-point Likert scale where higher values were synonymous with greater ease of accessibility.

Age, gender and nationality of participants were recorded using free text fields.

### Data analysis

Descriptive statistics and Mann-Whitney U tests (65) were used to compare mean values for perceived discrimination and devaluation, self-esteem, treatment-seeking threshold, and accessibility between psychiatric outpatient clinics that were located within the inner city or on the main site of the psychiatric university hospital at the outer city limits.

Shapiro-Wilk (66) and Levene (67) tests were conducted to assess normal distribution and variances, respectively. A Kruskal-Wallis test (68) was used to analyze differences based on the psychiatric service visited.

To examine the correlation between PDD, SE, and TST, Pearson’s correlation analysis was performed. Correlation analyses were calculated for the whole sample and for the subgroups of outpatient services located inside and outside the inner city limits. A significance level of *p* < 0.05 was used to determine the statistical significance of the correlation coefficient.

Partial correlations were performed to test whether gender, age and nationality of the subjects could be potential confounding variables. For the partial correlation analysis of nationality, participants’ countries of origin were grouped into regional categories due to some foreign nationalities being represented by one person. Nationality was used for descriptive statistics only because of uneven group sizes.

Confidence intervals with a coverage probability of 95% were calculated for the mean values and correlation coefficients to estimate the uncertainty surrounding the obtained results (69).

Any missing values were excluded pairwise. As this was an exploratory study aimed at generating new hypotheses, no correction for multiple testing was performed. Data were analyzed using IBM SPSS Statistics 27.0 (70).

## Results

**Table 1** provides information on the catchment area, type of contacts available, and spectrum of diagnoses treated in the different outpatient services. While most outpatient services have planned contacts, the walk-in outpatient service (inner city) and the patient admission/emergencies service (outer city) provide emergency services and unplanned appointments. Most services have the canton of Basel-Stadt and its surrounding regions as catchment area. The outpatient service for non-organic sleep disorders, however, serves a supracantonal catchment area.

**Table 1:** Psychiatric outpatient service characteristics and patient samples.

Psychiatric Outpatient Service	Catchment Area	Type of Contact (P/E)	Spectrum of Diagnoses (ICD-10)	Participants (*n* = 525, %)	Female gender (n, %)	Age (*m ± SD*)	Swiss nationality (n, %)	Participants who contacted the service themselves (n, %)
Outpatient Healthcare Center within the inner city limits (Kornhausgasse 7, CH-4051 Basel)								
Walk-in outpatient service	Cantonal	E	All	129 (24.6%)	77 (54.3%)	44.1 ± 15.9	96 (74.4%)	90 (69.8%)
Psychotic disorders	Cantonal	P	F2	69 (13.1%)	25 (36.2%)	48.6 ± 14.5	32 (46.4%)	22 (31.9%)
Transcultural psychiatry	Cantonal	P	F4	18 (3.4%)	7 (38.9%)	41.1 ± 12.3	1 (5.6%)	4 (22.2%)
ADHD and Asperger's	Cantonal	P	F9	21 (4.0%)	7 (33.3%)	37.1 ± 14.5	18 (85.7%)	10 (47.6%)
Substance use disorders	Cantonal	P	F1	10 (1.9%)	3 (30.0%)	47.5 ± 13.9	7 (70.0%)	8 (80.0%)
Gerontopsychiatry	Cantonal	P	All	57 (10.9%)	34 (59.6%)	75.2 ± 10.0	45 (78.9%)	20 (35.1%)
Outpatient treatment next to the University Hospital Basel (USB; Spitalstrasse 2, CH-4056 Basel)								
Heroin- assisted treatment	Cantonal	P	F1	28 (5.3%)	8 (28.6%)	50.1 ± 6.5	21 (75.0%)	19 (67.9%)
Outpatient treatment at the main site of the psychiatric university hospital at the outer city limits (Wilhelm Klein-Strasse 27, CH-4056 Basel)								
Patient admission/ emergencies	Cantonal	E	All	11 (2.1%)	4 (36.3%)	43.8 ± 15.0	5 (45.55%)	9 (81.8%)
Substitution treatment for substance use disorders	Cantonal	P	F1	28 (5.3%)	9 (32.1%)	49.6 ± 8.0	24 (85.7%)	20 (71.4%)
Behavioral addictions	Cantonal	P	F6	29 (5.5%)	8 (27.6%)	46.6 ± 14.6	22 (75.9%)	22 (75.9%)
CBT outpatient service	Cantonal	P	F4	89 (17.0%)	40 (44.9%)	44.2 ± 14.6	69 (77.5%)	57 (64.0%)
Non-organic sleep disorders	Supracantonal	P	F5	9 (1.7%)	4 (44.4%)	48.6 ± 13.5	7 (77.8%)	2 (22.2%)
Personality disorders	Cantonal	P	F6	13 (2.5%)	7 (53.8%)	32.6 ± 14.5	10 (76.9%)	5 (38.5%)
Privately insured patients	Cantonal	P	All	14 (2.7%)	8 (57.1%)	59.5 ± 15.4	14 (100.0%)	8 (57.1%)

P, planned contacts only; E, unplanned and emergency contacts; F, predominant diagnoses according to ICD-10 chapter F; m, mean; SD, standard deviation.

In addition, **Table 1** lists information on the number of participants from each outpatient service as well as descriptive statistics on gender, age, nationality and the percentage of participants who had contacted the service themselves. A final sample of 525 participants answered the survey, resulting in a response rate of 23.8% (for an overview on the distribution across outpatient services, please cf. **Table 1**). The response rates of the psychiatric outpatient clinics were at least 15.6% (substitution treatment for substance disorders) and a maximum of 42.4% (privately insured patients).

63.2% of the survey participants were patients from outpatient services located within the inner city, and 36.8% were from services located in the outer city limits. 44.6% of the participants were female. The participants were aged between 18 and 99 years with a mean (m) of 48.8, a standard deviation (SD) of 17.1 and a 95% confidence interval (CI) ranging from 47.2 to 50.3 years. 371 (70.7%) were Swiss citizens, 23 (4.4%) had the Swiss and a foreign nationality. Participants of foreign nationality were predominantly German (23; 4.4%), Italian (18; 3.4%), or Turkish (9; 1.7%). 8 (1.5%) participants were from other Western European countries, 13 (2.5%) from Eastern Europe and 1 (0.2%) participant was from mixed European descent. Outside of Europe, 7 (1.3%) participants were from Eastern Asia, 5 (1.0%) from the Middle East, 5 (1.0%) from African countries and 1 (0.2%) participant was from the USA. 41 (7.8%) participants did not specify their nationality.

296 (56.4%) participants had contacted the outpatient service themselves. 173 (33.0%) had started treatment in 2018. 106 (20.2%) out of 471 participants had already finished their treatment at the time of the survey, while 365 (69.5%) were still in treatment.

Descriptive statistics for perceived discrimination and devaluation (PDD), self-esteem (SE), treatment-seeking threshold (TST), and accessibility of each outpatient service are shown in **Table 2**.

**Table 2:** Clinical data of psychiatric services and perceived discrimination and devaluation (PDD), self-reported self-esteem (SE), threshold to seek treatment (TST) and accessibility of site by survey participants.

	PDD		SE		TST		Accessibility	
	*m ± SD*	*CI 95%*	*m ± SD*	*CI 95%*	*m ± SD*	*CI 95%*	*m ± SD*	*CI 95%*
**Total sample**	3.2 ± 0.8	3.15-3.29	4.4 ± 1.9	4.25-4.58	3.2 ± 2.3	2.95-3.37	6.1 ± 1.4	5.98-6.23
Outpatient Healthcare Center within the inner city limits (Kornhausgasse 7, CH-4051 Basel)								
Walk-in outpatient service	3.3 ± 0.8	3.12-3.39	4.3 ± 1.9	3.99-4.69	3.5 ± 2.3	3.08-3.90	6.5 ± 1.0	6.30-6.66
Psychotic disorders	3.2 ± 0.8	3.00-3.3.9	5.1 ± 2.1	4.57-5.64	3.2 ± 2.3	2.60-3.75	6.4 ± 1.2	6.15-6.73
Transcultural psychiatry	3.1 ± 1.0	2.62-3.61	5.7 ± 1.6	4.84-6.49	4.3 ± 2.5	3.08-5.50	6.7 ± 0.8	6.37-7.07
ADHD and Asperger's	3.2 ± 0.9	2.88-3.61	4.5 ± 1.4	3.82-5.08	2.6 ± 1.8	1.77-3.39	6.4 ± 1.0	5.96-6.80
Substance use disorders	3.0 ± 0.5	2.69-3.36	4.6 ± 2.4	3.02-6.09	3.3 ± 2.7	1.37-5.13	6.7 ± 1.0	6.11-7.29
Gerontopsychiatry	3.3 ± 0.8	3.13-3.57	4.4 ± 1.9	3.81-4.90	3.4 ± 2.6	2.63-4.20	6.3 ± 1.2	5.96-6.68
Outpatient treatment next to the University Hospital Basel (USB; Spitalstrasse 2, CH-4056 Basel)								
Heroin-assisted treatment	2.9 ± 0.7	2.61-3.13	4.3 ± 1.7	3.64-4.98	3.1 ± 2.1	2.24-3.92	6.5 ± 0.8	6.17-6.76
Outpatient treatment at the main site of the psychiatric university hospital at the outer city limits (Wilhelm Klein-Strasse 27, CH-4056 Basel)								
Patient admission/emergencies	2.8 ± 0.9	2.28-3.36	3.9 ± 1.6	2.91-4.89	4.2 ± 2.6	2.53-5.91	6.0 ± 1.4	5.02-6.89
Substitution treatment for substance use disorders	3.0 ± 0.8	2.69-3.34	4.3 ± 1.9	3.63-5.04	2.4 ± 2.1	1.51-3.19	5.6 ± 2.1	4.84-6.37
Behavioral addictions	3.4 ± 0.8	3.07-3.64	4.2 ± 1.5	3.57-4.73	3.4 ± 2.4	2.46-4.37	5.4 ±1.8	4.70-6.01
CBT outpatient service	3.3 ± 0.7	3.10-3.42	4.1 ± 1.8	3.71-4.45	2.8 ± 2.2	2.35-3.34	5.4 ± 1.7	5.08-5.80
Non-organic sleep disorders	3.3 ± 0.9	2.68-3.90	4.9 ± 1.4	3.94-5.81	2.3 ± 2.1	0.99-3.68	4.3 ± 2.1	2.78-5.72
Personality disorders	3.2 ± 0.8	2.69-3.63	3.8 ± 2.0	2.64-4.99	3.2 ± 1.9	2.07-4.30	5.8 ± 1.3	5.12-6.55
Privately insured patients	3.4 ± 0.7	3.03-3.76	4.5 ± 2.0	3.46-5.54	1.5 ± 0.9	0.98-1.94	6.2 ± 1.4	5.47-6.96

m, mean; SD, standard deviation; CI 95%, confidence interval of mean. Ratings on the Perceived Discrimination Devaluation Scale (PDD) were made on a 5-point Likert scale, with 1 indicating highest perceived discrimination and 5 indicating lowest perceived discrimination. Ratings for self-esteem (SE), treatment-seeking threshold (TST) and accessibility were made on a 7-point Likert scale with 1 indicating lowest and 7 indicating highest rating or approval.

In the total sample of *n* = 525, PDD was reported at a considerable level by all patients (mean of 3.2) with little variation (SD=0.8, CI 3.15-3.29). SE had a mean score of 4.4 ± 1.9 (CI 4.25-4.58). TST was generally low to medium with a mean of 3.2 ± 2.3 (CI 2.95-3.37). For all psychiatric services, accessibility was rated high with 6.1 ± 1.4 (CI 5.98-6.23).

Correlation analyses revealed a significant association between perceived discrimination and devaluation and self-esteem (*r* = 0.244, CI 0.156-0.329, *p* < 0.001) with a small effect size. Higher PDD scores indicating less stigma were associated with higher SE. Regarding TST, no significant correlation to PDD (*r* = -0.081, CI [-0.173] – [0.014], *p* = 0.093) or SE (*r* = -0.018, CI [-0.112] – [0.077], *p* = 0.712) was found in the total sample.

The data for SE, TST, and service location accessibility were not normally distributed (*p* < 0.001), while PDD was approximately normally distributed (*p* = 0.014). Levene’s test showed significant inequality of error variances (*p* < 0.001) when conducting an ANCOVA test with PDD as an independent variable. Due to violation of homogeneity of variances assumption, Kruskal-Wallis tests were used to analyze group differences among the 14 psychiatric outpatient services. Significant differences were found in SE (H(13) = 23,675, *p* = 0.034) and location accessibility (H(13) = 56,474, *p* < 0.001), but not in PDD (H(13) = 15,147, *p* = 0.298) or TST (H(13) = 21,574, *p* = 0.062). Further analysis was conducted through descriptive statistics and Mann-Whitney U tests.

346 participants from seven psychiatric outpatient services in the inner city, at either Kornhausgasse or Spitalstrasse, had an average PDD score of 3.2 (SD ± 0.8, CI 3.14-3.31), SE score of 4.6 ± 1.9 (CI 4.35-4.79), TST of 3.3 ± 2.3 (CI 3.01-3.53) and rated accessibility at 6.5 ± 1.1 (CI 6.33-6.57).

179 participants from seven outpatient services located at the psychiatric university hospital main site on the outer city limits had an average PDD score of 3.2 ± 0.8 (CI 3.09-3.33), SE score of 4.1 ± 1.7 (CI 3.88-4.40), TST score of 2.9 ± 2.2 (CI 2.58-3.29), and rated accessibility of treatment service at 5.4 ± 1.8 (CI 5.19-5.72).

Mann-Whitney U tests assessed differences in PDD, SE, TST, and accessibility of treatment location by outpatient service location. Patients at inner city services rated their accessibility significantly better than those treated at Wilhelm Klein-Strasse (*z* = -6.85, *p* < 0.001, *r* = 0.308). Inner city patients also had significantly higher SE (*z* = -2.62, *p* = 0.009, *r* = 0.123), but no significant differences were found in PDD (*z* = 0.19, *p* = 0.851) or TST (*z* = -1.39, *p* = 0.163) between groups. Positive correlations were found between PDD and SE for both inner city (*r* = 0.211, CI 0.099-0.318, *p* < 0.001) and Wilhelm Klein-Strasse (*r* = 0.314, CI 0.168-0.447, *p* < 0.001) patients. No significant correlations were found for TST and PDD (*r* = -0.051, CI [-0.213] – [0.114], *p* = 0.545) or SE (*r* = -0.111, CI [-0.267] – [0.050], *p* = 0.176) in either group.

The following analysis compares the ratings for PDD, SE, and TST for all 14 psychiatric outpatient services.

The highest levels of perceived stigma were reported by emergency patients (2.8 ± 0.9, CI 2.28-3.36) and patients with substance use disorders from three different outpatient services (2.9 ± 0.7, CI 2.61-3.13, 3.0 ± 0.5, CI 2.69-3.36 and 3.0 ± 0.8, CI 2.69-3.34) while patients from the behavioral addictions outpatient service (3.4 ± 0.8, CI 3.07-3.64) and privately insured patients (3.4 ± 0.7, CI 3.03-3.76) had the lowest PDD ratings.

Self-esteem ratings varied across services, with personality disorder, with patients using services for personality disorder (3.8 ± 2.0, CI 2.64-4.99), substance use disorder (4.3 ± 1.7, CI 3.64-4.98 and 4.3 ± 1.9, CI 3.63-5.04) and emergency services (3.9 ± 1.6, CI 2.91-4.89 and 4.3 ± 1.9, CI 3.99-4.69) having the lowest SE ratings. Patients from the transcultural psychiatry service (5.7 ± 1.6, CI 4.84-6.49), the psychosis outpatient service (5.1 ± 2.1, CI 4.57-5.64), and the outpatient service for non-organic sleep disorders (4.9 ± 1.4, CI 3.94-5.81) reported the highest SE ratings.

The lowest TST were reported by privately insured patients (1.5 ± 0.9, CI 0.98-1.94), patients from the service for non-organic sleep disorders (2.3 ± 2.1, CI 0.99-3.68), and patients receiving substitution treatment for substance use disorders (2.4 ± 2.1, CI 1.51-3.19). In addition, patients seeking diagnostic evaluation for ADHD and Asperger’s (2.6 ± 1.8, CI 1.77-3.39), and patients from the cognitive behavioral therapy outpatient service (2.8 ± 2.2, CI 2.35-3.34) had a relatively low TST. The highest TST were reported by emergency patients (4.2 ± 2.6, CI 2.53-5.91) at the main psychiatric hospital site and patients from the transcultural psychiatry service (4.3 ± 2.5, CI 3.08-5.50).

The emergency service at the main site of the clinic and the walk-in outpatient service in the inner city were compared with 12 services for planned contacts only using a Mann-Whitney U test. Significant differences were found for TST (*z* = [- 2.37], *p* = 0.018, *r* = 0.111) and accessibility (*z* = [- 2.90], *p* = 0.004, *r* = 0.131). Emergency patients showed a higher TST with a mean of 3.5 ± 2.3 (CI 3.1-3.9) in comparison to patients with planned appointments (3.0 ± 2.3, CI 2.8-3.3). The accessibility of the location was rated significantly better by emergency patients (6.5 ± 1.0, CI 6.3-6.6) than by patients in the planned setting (6.0 ± 1.5, CI 5.8-6.1). No significant differences emerged for PDD (*z* = [- 0.46], *p* = 0.643) and SE (*z* = [- 0.78], *p* = 0.434).

Partial correlations with gender, age distribution and nationality as possible confounding variables showed no change in effect size or direction of the correlations between perceived stigma and self-esteem.

Gender and age were tested for bivariate correlations with PDD, SE and TST. Between the age of the participants and TST, there was a significant negative relationship (*r* = -0.111, CI [-0.203] - [-0.016], *p* = 0.022). Gender was not significantly related to any of the examined variables.

## Discussion

This study examined the association of outpatient services location with patients’ self-reported perceived stigma, self-esteem, treatment-seeking threshold, and service accessibility, and explored the association between perceived stigma, self-esteem, and treatment-seeking threshold in psychiatric outpatients. The adequately sized survey sample stems from a large university psychiatry tertiary care service provider covering all adult psychiatric diagnosis groups. Outpatient services ranged from emergency services and specialized outpatient services for specific diagnosis or age groups to outpatient services dedicated to privately insured patients. Half of the services were from outpatient services located in the inner city (with about two thirds of the participants), half were located at the outer city limit at the location of the main psychiatric hospital (with about one third of the participants). Thus, the current survey covered a broad and diverse range of outpatient services and allowed for a comparison of two geographical locations.

Concerning the relationship between perceived stigma, self-esteem, and treatment-seeking threshold, we found a positive correlation between low perceived discrimination/devaluation and self-esteem, which is in accordance with the published literature (15, 29, 71, 72). However, no significant correlations between perceived stigmatization and treatment-seeking threshold emerged in our sample. In addition, no significant correlations between self-esteem and treatment-seeking threshold were found. While this result certainly has to be replicated in future studies, it indicates that other factors might be relevant for the threshold to seek treatment. According to the majority of empirical evidence, both perceived and self-stigma is associated with a negative attitude towards treatment (73–75). However, the systematic review and meta-analysis by Schnyder and colleagues (2017) found that participants’ own negative attitudes toward seeking mental health help and their stigmatizing attitudes toward people with mental illness were stronger associated with lower active help-seeking whereas perceived stigma did not prove to be a significant predictor (76). Other studies that focused on the role of perceived stigma also failed to demonstrate its influence as a correlate of treatment seeking (77). Instead, other factors like lack of knowledge about mental illnesses and their treatments, negative attitudes toward mental health treatment and a preference for self-reliance might have a greater influence on treatment uptake (23, 31). In their study on individuals with major depression, the research group of Schomerus (2013) found that older age, higher education level, high conscientiousness, low resilience, social support, childhood abuse, and more severe depression were linked with help-seeking (78). In our study, we identified the type of contact as a potential factor influencing the treatment-seeking threshold, with significantly higher treatment-seeking thresholds observed in emergency patients compared to patients with scheduled appointments. This is consistent with the findings of negative stereotypes and higher stigma toward psychiatric emergency departments. Increasing knowledge on the topic of treatment-seeking threshold might mean having a possibility to reduce this threshold and help getting more persons in need into treatment.

In regards to the impact of location of psychiatric outpatient treatment sites on perceived stigma, self-esteem, treatment-seeking threshold and accessibility from the service user’s perspective, significant differences were discovered in favor of the inner city services for self-esteem and accessibility.

When comparing the services at both locations separately, it was found that the accessibility ratings for services in the inner city were relatively similar, while those on the outer city limit exhibited greater variance. The highest rating for accessibility for the psychiatric services at the outer city limits came from privately insured patients and was only slightly lower than the accessibility of the seven services in the city center. For these patients, being located in a more remote location might even be considered as an advantage to maintain privacy. Conversely, the non-organic sleep disorder service with a larger catchment area received the lowest accessibility ratings, which may correspond to the longer distances patients have to take into account to reach the service from their place of living.

The comparison between services for unplanned emergencies versus planned contact only unveiled distinct differences between location accessibility and the treatment-seeking threshold. Emergency services, where reaching the location quickly may not be a priority due to the urgent nature of the mental health issue, were rated highly in terms of accessibility. This outcome could potentially indicate lower expectations from emergency patients or higher demands from patients with scheduled appointments. It is plausible that patients with scheduled appointments may receive treatment over an extended period, making the accessibility of the location a more crucial factor than for a one-time appointment. The threshold for seeking treatment was found to be significantly higher for emergency patients, particularly for those seeking help at the main psychiatric hospital site. These pronounced inhibitions among emergency patients align with prior research (35).

In relation to the impact of treatment focus of the psychiatric service, only self-esteem and accessibility showed significant variation. Here, the findings were different for the individual outpatient services, with patients receiving treatment at emergency services, services for substance use disorder and services aimed at personality disorders having the lowest ratings for self-esteem. This is in line with the existing literature (35, 43, 79). Patients from the outpatient service for non-organic sleep disorders had a relatively high self-esteem, which corresponds to low ratings for perceived stigma and to the relatively good acceptance of this field of mental health problems in society (80, 81). However, and contra intuitively (36, 39, 82, 83), patients from the psychosis outpatient service and from the transcultural psychiatry service reported the highest self-esteem ratings. In line with the aforementioned studies, we would have expected lower self-esteem in patients who are or have been treated in the transcultural psychiatric services and in the service specialized on psychosis treatment. We speculate that in these services, there might have been a response bias filtering out the more impaired patients with potentially lower self-esteem.

Lastly, there were no significant differences between inner city and outer city services regarding perceived discrimination and threshold to seek treatment. Perceived discrimination was considerable for all patients with little variation. Patients receiving treatment from the behavioral addictions outpatient service and privately insured patients reported the least perceived stigmatization, while emergency patients at the site of the psychiatric hospital and patients with substance use disorders rated perceived stigma highest. This is also compatible with the published literature (35, 84, 85). The threshold to seek treatment varied from low to medium, with privately insured patients, those from the cognitive-behavioral therapy (CBT) outpatient (86, 87), patients with non-organic sleep disorders (81), and those seeking diagnostic evaluation of ADHD and Asperger’s (88, 89) reported the lowest treatment-seeking thresholds.

Contrary to the established pattern of SUD services having patients with the highest perceived stigmatization and lowest self-esteem, patients receiving substitution treatment for substance use disorders reported a relatively low threshold to seek treatment. This patient group tends to have a long-standing therapeutic relationship with multiple daily visits to the outpatient service to receive their medication, which might help explain this finding. Aside from emergency patients, the highest treatment-seeking threshold was reported by patients of the transcultural psychiatry service. In contrast to the high self-esteem, the high treatment-seeking threshold of patients in the transcultural outpatient service is consistent with previous findings (55, 57, 90, 91). Probable explanations for the increased treatment-seeking threshold in transcultural outpatients could be explained, among other things, by language barriers, and depending on the cultural background, by negative stereotypes of mental illness and psychiatric treatment and preference for help from family members or traditional health services.

Regarding potential confounding variables, such as gender, age, and nationality, we did not find the effect of gender found in other studies, according to which men and women differ in their help-seeking behavior for mental problems by public stigma and self-stigma, in the present sample. We also found no evidence of increased treatment inhibition among males in the gender distribution of the study participants. In our sample, the gender ratios were almost equally distributed. This means that about as many men as women sought therapeutic support in the 14 psychiatric outpatient clinics of the UPK Basel and took part in our patient survey. On the other hand, not all studies found gender differences for the perception of stigma (92).

Age seemed to have a significant influence and their perception and handling of barriers towards treatment seeking. This is consistent with other research that has also found lower inhibitions to therapeutic treatment in older individuals (54).

Nationality did not change the important link between stigma and self-esteem in the actual correlation. However, the variable was not appropriate for a bivariate correlation with stigma, self-esteem, and threshold due to the significantly different group sizes and low representation of ethnic minorities. Although nationality was recorded in our sample, no information was provided on the countries where the participants had resided in their lifetime, and thus it is unclear which cultures may have influenced them. Cultural factors other than nationality that could potentially influence perceived stigma and treatment-seeking threshold were not part of our study and could be subject of future research. These factors could include cultural differences in the frequency and prevalence of mental illnesses, perception and expression of mental illness symptoms, understanding of mental illness treatment, attitudes towards traditional and alternative treatment approaches, religious and spiritual beliefs, and coping with mentally ill family members (57, 91, 93).

### Limitations

It is important to acknowledge certain limitations of this study. First, although the total sample size of 525 participants is adequate for our analyses, our survey may be underpowered to detect differences between outpatient services with small participant numbers. In addition, even with an adequate response rate of about 24%, a large percentage of patients did not take part in this survey. Especially patients with high perceived discrimination and low self-esteem and individuals with a high treatment-seeking threshold might not have responded. Directly examining patients during their stay at the outpatient service could help to avoid this limitation in future research, though some patients may still choose not to answer questionnaires.

The low number of cases in some outpatient clinics impairs the statistical calculations and interpretability of the results. The width of the confidence intervals indicates which mean values should be interpreted with caution.

In addition, the use of self-report measures introduces potential biases due to social desirability and individual differences in introspection. Self-esteem, threshold to seek treatment, and service accessibility were each surveyed using only a single item (33). Although this is intended to ensure the cost- and time-effectiveness of the questionnaire, it may mean that not all aspects of these complex constructs were mapped. The application of a single item self-esteem scale has, however, been shown to be a valid, reliable, and economical instrument (94, 95).

Clinical data like patient’s diagnosis, severity and course of illness were not available. Therefore, the treatment focus of the outpatient clinic had to be used as a proxy.

We only performed analyses using the total PDD score and did not examine the individual twelve items of the PDD scale. It is possible that certain items, such as agreeing to let mentally ill individuals care for children, employment opportunities, or challenges in forming and maintaining friendships, may have been rated differently and correlated differently with self-esteem and treatment-seeking threshold.

The sample primarily included Swiss citizens and residents of other European countries. The participants’ nationality was only used in the descriptive statistics and partial correlation analysis due to the uneven distribution of groups. While the Swiss group was sizable with 371 individuals, the groups of participants from other nationalities, typically consisting of fewer than 20 people per country or region, were too small to draw conclusive findings.

The study only contains answers of people with mental illnesses who sought help and in that process found access to the treatment site or might have overcome inhibitions towards treatment. There is no information on the general population’s perception of accessibility of treatment at the mentioned treatment sites nor on people with mental illnesses who did not find access to treatment. It would be interesting to compare the values of the surveyed sample, particularly self-esteem and perceived stigma, with a population without mental illness.

### Conclusion and recommendations for future research

We showed significant correlation of perceived discrimination and devaluation and self-esteem, whereas there was no significant association between these variables with threshold to seek treatment. It was evident that many patients experienced stigmatization, impaired self-esteem, underlining the importance of research in this area. On the other hand, threshold to seek treatment was reported low to medium in the whole sample, with patients from some outpatient services reporting higher thresholds.

For future research, it would be beneficial to explore various forms of stigma, and to incorporate longitudinal research methods. It is recommended to consider temporal criteria, such as the frequency and number of appointments at psychiatric outpatient services, in future surveys. Additionally, studying whether perceived stigma, treatment-seeking threshold, and accessibility to locations are influenced by the frequency of appointments at outpatient clinics could provide valuable insights.

Previous studies indicate that individuals’ own stereotypes and negative attitudes towards mental health treatment may have a stronger influence on the decision to seek treatment than perceived stigma. Therefore, it is important to include these variables in future studies. Factors like socio-economic status, employment status, and education level of participants could help explain variations in assessments of stigma, self-esteem, and treatment-seeking threshold.

Future research should consider potential confounders that may affect the outcomes being studied. For instance, treatment satisfaction was not taken into account in the analysis of this study. Given that treatment satisfaction can impact perceived stigma, self-esteem, and treatment-seeking threshold (96–98), further studies could focus on patients’ evaluations of treatment and their interactions with the variables studied here. Investigating whether factors that improve treatment satisfaction like treatment type, care continuity and involvement of patients (98), influence stigmatization and enhance the willingness to seek treatment, could be of interest. On the other hand, factors that diminish treatment satisfaction like issues with finances and accommodation, physical health needs and a high percentage of occupied beds (98) could also lead to an increase in self-stigma and reduce motivation for help-seeking in the future.

Other factors such as diagnosis, illness severity, coping mechanisms, social support, personality traits, and cultural aspects should also be explored for their associations with perceived stigmatization and treatment-seeking threshold.

Differences in accessibility were mainly linked to location, with outpatient services in inner cities being significantly more accessible. However, the perception of accessibility also depends on the needs of patient groups addressed. These results should remind authorities responsible for planning mental health services that it is important to lower the treatment-seeking threshold to seek treatment and to focus on the service users’ perspective.

## Data availability statement

The raw data supporting the conclusions of this article will be made available by the authors, without undue reservation.

## Ethics statement

The studies involving humans were approved by Ethics Committee of Northwestern Switzerland. The studies were conducted in accordance with the local legislation and institutional requirements. Written informed consent for participation was not required from the participants or the participants’ legal guardians/next of kin in accordance with the national legislation and institutional requirements.

## Author contributions

JB: Formal analysis, Writing – original draft. LI: Writing – review & editing. CR: Writing – review & editing. JM: Writing – review & editing. UL: Writing – review & editing. CH: Formal analysis, Writing – original draft.
